# Digital Clubbing Is Associated with Higher Serum KL-6 Levels and Lower Pulmonary Function in Patients with Interstitial Lung Disease

**DOI:** 10.1155/2018/3640967

**Published:** 2018-01-23

**Authors:** Kazushige Shiraishi, Torahiko Jinta, Naoki Nishimura, Hiroshi Nakaoka, Ryosuke Tsugitomi, Kohei Okafuji, Atsushi Kitamura, Yutaka Tomishima, Gautam A. Deshpande, Tomohide Tamura

**Affiliations:** ^1^Department of Pulmonary Medicine, St. Luke's International University, St. Luke's International Hospital, Tokyo, Japan; ^2^Center for Clinical Epidemiology, St. Luke's International University, St. Luke's International Hospital, Tokyo, Japan

## Abstract

**Background:**

Although digital clubbing is a common presentation in patients with interstitial lung disease (ILD), little has been reported regarding its role in assessing patients with ILD. This study evaluated patients with ILD for the presence of clubbing and investigated its association with clinical data.

**Methods:**

We evaluated patients with ILD who visited the teaching hospital at which the study was conducted, between October 2014 and January 2015. Clubbing, evaluated using a Vernier caliper for individual patients, was defined as a phalangeal depth ratio > 1. We examined the association of clubbing with clinical data.

**Results:**

Of 102 patients with ILD, we identified 17 (16.7%) with clubbing. The partial pressure of oxygen in arterial blood was lower (65.2 ± 5.9 mmHg versus 80.2 ± 3.1 mmHg; *p*=0.03), serum Krebs von den Lugen-6 (KL-6) levels were higher (1495.0 ± 277.4 U/mL versus 839.1 ± 70.2 U/mL; *p*=0.001), and the percent predicted diffusing capacity of carbon monoxide was lower (50.0 ± 6.0 versus 73.5 ± 3.1; *p*=0.002) in these patients with clubbing.

**Conclusions:**

Patients with clubbing had lower oxygen levels, higher serum KL-6 levels, and lower pulmonary function than those without clubbing.

## 1. Introduction

Interstitial lung disease (ILD) is a heterogeneous cluster of pulmonary disorders characterized by diffuse parenchymal lung damage [1]. Disease activity in ILD is objectively assessed by pulmonary function testing, chest radiography, and high-resolution computed tomography (HRCT) [1]. Serum Krebs von den Lugen-6 (KL-6) is a biomarker for ILD [2] and can be used to evaluate disease activity in ILD and to predict clinical outcomes [3].

Digital clubbing was first recognized as a sign of empyema by Hippocrates in the fifth century BC [4]. Studied for centuries, clubbing has been associated with a variety of pulmonary diseases, including infections, ILD, and neoplasms [5]. In the clinic setting, the skilled primary care physician and pulmonology specialist may search for clubbing to identify patients with underlying chronic lung disease. However, little has been reported regarding the value of clubbing when assessing patients with ILD. As such, we carried out a cross-sectional assessment of patients with ILD for the presence of clubbing and investigated its association with clinical data, including blood oxygenation measurements, serum KL-6 levels, and pulmonary function test results.

## 2. Methods

### 2.1. Patients and Study Design

This cross-sectional retrospective study examined adult outpatients with a diagnosis of ILD visiting the Pulmonology Department at St. Luke's International Hospital (Tokyo, Japan) between October 2014 and January 2015. Patients with a history of diseases shown to be associated with clubbing, including lung carcinoma, asbestosis, active tuberculosis, and sarcoidosis [5], were excluded. Each participant was assessed for clubbing after which the medical charts of participants were reviewed and the following data were extracted: age, sex, pack-years of tobacco smoking, systolic pulmonary arterial pressure (sPAP), and left ventricular ejection fraction (LVEF) estimated by Doppler echocardiography, pulse oximeter oxygen saturation (SpO_2_), arterial blood gas analysis results, serum KL-6 levels, pulmonary function test results, etiology of ILD, and HRCT imaging patterns of ILD as assessed by radiologists. ILD-GAP index was calculated using the clinical data [6].

### 2.2. Definition of Clubbing

The presence of clubbing was assessed by objective measurement of a phalangeal depth ratio, calculated by distal phalangeal finger depth (DPD) divided by interphalangeal finger depth (IPD), with the use of a Vernier caliper [7]. Clubbing was defined as the phalangeal depth ratio greater than 1. While there are many techniques available for assessment of clubbing including profile sign, hyponychial angle, and Schamroth sign [4], Myers and Farquhar recommend in their systematic review that the phalangeal depth ratio be used for the objective and quantitative assessment of clubbing [4]. A phalangeal depth ratio above 1 is rare in disease-free subjects and was reported to be independent of race, age, and sex [4]. Technically, the caliper was gently placed on an index finger, and distal/interphalangeal finger depth was measured without pressing the tissue [7].

### 2.3. Statistical Analysis

Categorical variables were compared using Fisher's exact test, continuous variables were compared using the unpaired *t*-test, and binomial variables were compared using Fisher's exact test. All statistical analyses were performed using SPSS version 21.0 (SPSS Inc., Armonk, NY, USA). A *p* value < 0.05 was considered to be statistically significant.

### 2.4. Ethical Approval

This study was conducted in accordance with the amended Declaration of Helsinki. Ethical approval was provided by the Ethics Committee of St. Luke's International Hospital, and individual participant consent was obtained.

## 3. Results

### 3.1. Study Participants

A total of 102 patients with HRCT-confirmed ILD met the inclusion criteria, of whom 17 (16.7%) patients had clubbing.  Table 1 shows the clinical and demographic characteristics of patients with ILD. Age, sex, pack-years of smoking, sPAP, LVEF, and HRCT imaging patterns were not significantly different between the two groups. The diagnostic category of ILD, based on the presence of connective tissue disease (CTD) and the high-resolution CT (HRCT) imaging pattern, is shown in Table 2. All patients with connective tissue disease (CTD) had their diagnoses confirmed by an immunology/rheumatology specialist, and a total of 50 patients with connective tissue disease-associated interstitial lung disease (CTD-ILD) were observed. The HRCT imaging pattern was dependent on the radiologists' clinical decision, in accordance with international guidelines [8]. The prevalence of clubbing in relation to each diagnostic category (Table 2) was as follows: non-CTD-ILD with radiological nonspecific interstitial pneumonia (NSIP) pattern, 20.8% (*N* = 5); CTD-ILD, 12.0% (*N* = 6); rheumatoid arthritis-associated interstitial lung disease (RA-ILD), 6.7% (*N* = 1); and systemic sclerosis-associated interstitial lung disease (SSc-ILD), 25.0% (*N* = 5). No clubbing was observed in patients with dermatomyositis-associated interstitial lung disease (DM-ILD) and Sjogren's syndrome-associated interstitial lung disease (SS-ILD).

### 3.2. Blood Oxygenation Measurements, Serum KL-6, and Pulmonary Function

Table 3 summarizes SpO_2_, arterial blood gas results, serum KL-6 levels, and pulmonary function test results for patients with and without clubbing. The following blood oxygenation measurements were significantly lower in those with clubbing: SpO_2_ (92.7 ± 1.2 (*N* = 17) versus 96.0 ± 0.3 (*N* = 78); *p* < 0.001) and the partial pressure of oxygen in arterial blood (PaO_2_; 65.2 ± 5.9% (*N* = 6) versus 80.2 ± 3.1 mmHg (*N* = 23); *p*=0.033). Serum KL-6 was significantly higher in patients with clubbing (1495.0 ± 277.4 U/mL (*N* = 14) versus 839.1 ± 70.2 U/mL (*N* = 82); *p*=0.001). The following pulmonary function test results were significantly lower in those with clubbing: %FEV_1_ (82.2 ± 4.7 (*N* = 15) versus 98.9 ± 2.9 (*N* = 63); *p*=0.01); %FVC (77.7 ± 3.5 (*N* = 15) versus 94.3 ± 2.7 (*N* = 63); *p*=0.006); %VC (78.6 ± 3.5 (*N* = 15) versus 96.3 ± 2.7 (*N* = 63); *p*=0.003); and %DLCO (50.0 ± 6.0 (*N* = 12) versus 73.5 ± 3.1 (*N* = 57); *p*=0.002). Although the difference was not significant, the ILD-GAP index was higher in patients with clubbing (2.17 ± 0.5 U/mL (*N* = 12) versus 1.28 ± 0.3 U/mL (*N* = 57); *p*=0.154).

Similarly, SpO_2_ was significantly lower in patients with clubbing in the CTD-ILD group (93 ± 2.5 (*N* = 6) versus 96.6 ± 0.3 (*N* = 38); *p*=0.004), serum KL-6 was significantly higher (2262 ± 547.4 U/mL (*N* = 6) versus 969.7 ± 108.2 U/mL (*N* = 43); *p*=0.0006), and pulmonary function test results were significantly lower in those with clubbing (%DLCO: 45.3 ± 4.1 (*N* = 6) versus 73.5 ± 4.2 (*N* = 33); *p*=0.008). Although not significant, the same trend was observed in the non-CTD-ILD NSIP group. PaO_2_ values for the CTD-ILD group and the non-CTD-ILD NSIP group are not shown because the numbers of measurements obtained were very small.

## 4. Discussion

In this study, we showed that the presence of clubbing in patients with ILD was associated with lower blood oxygenation levels, higher serum KL-6 levels, and lower pulmonary function when compared to those without clubbing, regardless of the underlying etiology of ILD. Because lower blood oxygenation levels and low pulmonary function are seen in patients with advanced ILD, our results suggest that clubbing may be associated with disease progression. Higher serum KL-6 levels in patients with clubbing also suggest that ILD disease activity may also be associated with the presence of clubbing. In concordance with these findings, the ILD-GAP score was higher in patients with clubbing, suggesting that the presence of clubbing could also be associated with poor prognosis.

We also found that the prevalence of clubbing in patients with radiological NSIP pattern, RA-ILD, and SSc-ILD was 20.8%, 6.7%, and 25%, respectively. To our knowledge, this study is the first to report the prevalence of clubbing in SSc-ILD. The prevalence of clubbing in NSIP and RA-ILD was consistent with that in previous reports [9–11]. We did not find any patient with clubbing in those with DM-ILD, which is consistent with previous reports of clubbing being rare in DM-ILD [12]. We confirmed that clubbing is indeed uncommon in DM-ILD; this result would suggest that clubbing in patients with DM-ILD may indicate the presence of an occult pathology, such as lung carcinoma or other CTD. Ishioka et al. reported that four out of 33 patients with CTD-ILD had clubbing, and the prevalence of clubbing in CTD-ILD was significantly lower than in patients with idiopathic interstitial pneumonia (16 out of 44 patients) [13]. Similarly, the prevalence of clubbing in patients with CTD-ILD (12.0%) was lower than in patients with non-CTD-ILD (21.1%) in the current study. There may be specific cellular or molecular mechanisms underlying CTD-ILD that suppress the emergence of clubbing, or there may be mechanisms in non-CTD-ILD that promote the emergence of clubbing. Further research on the pathophysiology of clubbing is needed to explain the difference.

We found that hypoxia is associated with clubbing in patients with ILD, and we presume from this that hypoxia could be responsible, to some extent, for clubbing in the study population. Several mechanisms including hypoxia and growth factors have been proposed for the pathogenesis of clubbing; however, the true mechanism of pathogenesis remains elusive [5]. Uppal et al. found that 15-hydroxyprostaglandin dehydrogenase, the enzyme-mediating prostaglandin degradation, is responsible for familial cases of clubbing and hypertrophic osteoarthropathy [14]. They also showed that elevated prostaglandin E2 (PGE2) levels were found in homozygous familial cases [14]. Their report suggests that clubbing may be mediated by PGE2. In accordance with that theory, Kozak et al. reported that clubbing in patients with lung cancer was associated with elevated urinary levels of PGE2 [15]. Cyclooxygenase-derived prostanoids are reported to be important mediators that regulate pulmonary function in normal and pathological conditions, and with regard to the relationship between PGE2 and ILD, PGE2 has been shown to be a key factor that controls fibroblast differentiation and proliferation in mouse models of pulmonary fibrosis [16]. In addition, cyclooxygenase-2, which induces PGE2, is reported to be widely expressed in the epithelium of patients with idiopathic pulmonary fibrosis (IPF), asbestosis, or cryptogenic organizing pneumonia [17]. Given these findings, measuring serum PGE2 levels in patients with ILD who have clubbing might be an interesting area of future research, although other factors, including hypoxia, growth hormones, platelet-derived growth factor, and vascular endothelial growth factor, have also been discussed in relation to the pathogenesis of clubbing [5].

This study has some limitations. First, many data were missing in the pulmonary function tests, which could have biased the results. However, missing data were mostly in the clubbing (−) population, and the patients without pulmonary function test data were clinically stable. That is, the results might have favored the clubbing (+) population. Second, radiographic quantification and histopathological assessment of ILD were not performed. In addition, although 52 non-CTD-ILD patients were identified in the study, patients with IPF or hypersensitivity pneumonitis were not included. This was due to difficulties in the diagnosis of interstitial pneumonia, the short study period, and the retrospective nature of the study. Lastly, the present study was a single-center, cross-sectional study, and the number of outcomes was not enough for adequate power in some analyses.

## 5. Conclusions

In conclusion, patients with clubbing showed lower blood oxygenation levels, higher serum KL-6 levels, and lower pulmonary function when compared with patients without clubbing, regardless of the underlying etiology of ILD. The results suggest that clubbing may be associated with disease progression, disease activity, and prognosis in ILD.

## Figures and Tables

**Table 1:** Characteristics of patients.

Characteristic	Clubbing (−)	Clubbing (+)	*p* value
	*n* = 85	*n* = 17	
Age, years	70.9 ± 12.87	64.2 ± 13.6	0.06
Male sex, number	48 (65)	9 (64)	0.79
Pack-years	26.4 ± 29.2	28.2 ± 40.5	0.83
sPAP, mmHg^†^	30.9 ± 13.0	33.8 ± 14.6	0.49
LVEF, %^†^	62.0 ± 9.7	60.5 ± 9.5	0.61
HRCT imaging patterns			
NSIP	47 (55.3%)	10 (58.8%)	1.00
UIP	12 (14.1%)	2 (11.8%)	1.00
OP	3 (3.5%)	0 (0%)	1.00
Pattern not specified	23 (27.1%)	5 (29.4%)	0.84

*Note.* Data are means ± standard deviation or number (%); sPAP = systolic pulmonary arterial pressure; LVEF = left ventricular ejection fraction; ^†^data were missing for 33 patients in the clubbing (−) group and four patients in the clubbing (+) group for sPAP and 24 patients in the clubbing (−) group and three patients in the clubbing (+) group for LVEF.

**Table 2:** Diagnostic category of participants.

Diagnostic category	Clubbing (−)	Clubbing (+)	*p* value	Prevalence of clubbing
	*n* = 85	*n* = 17		
CTD-ILD	44 (51.8%)	6 (35.3%)	0.29	12.0%
RA-ILD	14 (16.5%)	1 (5.9%)	0.46	6.7%
NSIP pattern	7	1	—	—
UIP pattern	3	0	—	—
Pattern not specified	4	0	—	—
DM-ILD	12 (14.1%)	0 (0%)	1	0.0%
NSIP pattern	12	0	—	—
SSc-ILD	15 (17.6%)	5 (29.4%)	0.32	25.0%
NSIP pattern	8	4	—	—
UIP pattern	1	0	—	—
OP pattern	1	0	—	—
Pattern not specified	5	1	—	—
SS-ILD	3 (3.5%)	0 (0%)	1	0.0%
NSIP pattern	1	0	—	—
UIP pattern	1	0	—	—
OP pattern	1	0	—	—
Non-CTD-ILD	41 (48.2%)	11 (64.7%)	0.29	21.1%
NSIP pattern	19 (22.4%)	5 (29.4%)	0.54	20.8%
HP suspected	1	1	—	—
IIP	0	1	—	—
UIP pattern	7 (8.2%)	2 (11.8%)	0.64	22.2%
HP suspected	1	0	—	—
OP pattern	1 (1.2%)	0 (0%)	1	0.0%
Pattern not specified	14 (16.5%)	4 (23.5%)	0.49	22.2%

CTD-ILD = connective tissue disease-associated interstitial lung disease; RA = rheumatoid arthritis; NSIP = nonspecific interstitial pneumonia; UIP = usual interstitial pneumonia; DM = dermatomyositis; SSc = systemic sclerosis; OP = organizing pneumonia; SS = Sjogren's disease; HP = hypersensitivity pneumonia; IIP = idiopathic interstitial pneumonia.

**Table 3:** Clinical data analysis between patients with and without clubbing.

Variable	Clubbing (−)	Clubbing (+)	*p* value
All patients with ILD^†^	*n* = 85	*n* = 17	—
SpO_2_, %	96.0 ± 0.3	92.7 ± 1.2	<0.001
PaO_2_, mmHg	80.2 ± 3.1	65.2 ± 5.9	0.033
PaCO_2_, mmHg	36.8 ± 1.1	39.5 ± 1.6	0.263
KL-6, U/mL	839 ± 70	1495 ± 277	0.001
%FEV1	98.9 ± 2.9	82.2 ± 4.7	0.010
%FVC	94.3 ± 2.7	77.7 ± 3.5	0.006
%VC	96.3 ± 2.7	78.6 ± 3.5	0.003
%DLCO	73.5 ± 3.1	50.0 ± 6.0	0.002
ILD-GAP index	1.28 ± 0.3	2.17 ± 0.5	0.154
CTD-ILD^‡^	*n* = 44	*n* = 6	—
SpO_2_, %	96.6 ± 0.3	93 ± 2.5	0.004
KL-6, U/mL	970 ± 108	2262 ± 547	0.001
%FEV1	94.8 ± 3.5	78.7 ± 9.9	0.09
%FVC	91.3 ± 3.0	73.8 ± 8.3	0.04
%VC	94.0 ± 2.9	75.5 ± 8.3	0.02
%DLCO	73.5 ± 4.2	45.3 ± 4.1	0.008
ILD-GAP index	0.03 ± 0.2	0.83 ± 0.6	0.208
Non-CTD-ILD, NSIP pattern^§^	*n* = 19	*n* = 5	—
SpO_2_, %	96.0 ± 0.3	95 ± 0.5	0.173
KL-6, U/mL	669 ± 66	1078 ± 390	0.078
%FEV1	101.7 ± 4.7	82.8 ± 8.6	0.083
%FVC	94.3 ± 2.7	77.7 ± 3.5	0.006
%VC	95.4 ± 6.8	80.9 ± 3.1	0.297
%DLCO	77.0 ± 6.5	53.0 ± 14.8	0.129
ILD-GAP index	3.00 ± 0.3	3.00 ± 0.6	1.000

*Note.* Data are means ± standard error; SpO_2_ = pulse oximeter oxygen saturation; PaO_2_ = partial pressure of oxygen in arterial blood; PaCO_2_ = partial pressure of carbon dioxide in arterial blood; ILD = interstitial lung disease; KL-6 = Krebs von den Lugen-6; FEV1 = forced expiratory volume in 1 second; FVC = forced vital capacity; VC = vital capacity; DLCO = diffusing capacity of carbon monoxide; NSIP = nonspecific interstitial pneumonia; CTD-ILD = connective tissue disease-associated interstitial lung disease; ^†^data were missing for eight patients in the clubbing (−) group for SpO_2_; 62 patients in the clubbing (−) group and 11 patients in the clubbing (−) group for PaO_2_ and PaCO_2_, respectively; three patients in the clubbing (−) group and one patient in the clubbing (+) group for KL-6; 22 patients in the clubbing (−) group and two patients in the clubbing (+) group for %FEV1, %FVC, and %VC; 28 patients in the clubbing (−) group and five patients in the clubbing (+) group for %DLCO and ILD-GAP index; ^‡^data were missing for six patients in the clubbing (−) group for SpO_2_; one patient in the clubbing (−) group for KL-6; nine patients in the clubbing (−) group for %FEV1, %FVC, and %VC; and 11 patients in the clubbing (−) group for %DLCO and ILD-GAP index; ^§^data were missing for one patient in the clubbing (−) group for KL-6; four patients in the clubbing (−) group for %FEV1, %FVC, and %VC; and one patient in the clubbing (−) group for %DLCO and ILD-GAP index.

## References

[1] Mathai S. C., Danoff S. K. (2016). Management of interstitial lung disease associated with connective tissue disease. *BMJ*.

[2] Kobayashi J., Kitamura S. (1995). KL-6: a serum marker for interstitial pneumonia. *Chest*.

[3] Ishikawa N., Hattori N., Yokoyama A., Kohno N. (2012). Utility of KL-6/MUC1 in the clinical management of interstitial lung diseases. *Respiratory Investigation*.

[4] Myers K. A., Farquhar D. R. (2001). The rational clinical examination. Does this patient have clubbing?. *JAMA*.

[5] Spicknall K. E., Zirwas M. J., English J. C. (2005). Clubbing: an update on diagnosis, differential diagnosis, pathophysiology, and clinical relevance. *Journal of the American Academy of Dermatology*.

[6] Ryerson C. J., Vittinghoff E., Ley B. (2014). Predicting survival across chronic interstitial lung disease: the ILD-GAP model. *Chest*.

[7] Baughman R. P., Gunther K. L., Buchsbaum J. A., Lower E. E. (1998). Prevalence of digital clubbing in bronchogenic carcinoma by a new digital index. *Clinical and Experimental Rheumatology*.

[8] Raghu G., Collard H. R., Egan J. J. (2011). An official ATS/ERS/JRS/ALAT statement: idiopathic pulmonary fibrosis: evidence-based guidelines for diagnosis and management. *American Journal of Respiratory and Critical Care Medicine*.

[9] McDonagh J., Greaves M., Wright A. R., Heycock C., Owen J. P., Kelly C. (1994). High resolution computed tomography of the lungs in patients with rheumatoid arthritis and interstitial lung disease. *British Journal of Rheumatology*.

[10] Rajasekaran B. A., Shovlin D., Lord P., Kelly C. A. (2001). Interstitial lung disease in patients with rheumatoid arthritis: a comparison with cryptogenic fibrosing alveolitis. *Rheumatology*.

[11] Li X., Chen C., Xu J. (2014). Nonspecific interstitial pneumonia and usual interstitial pneumonia: comparison of the clinicopathologic features and prognosis. *Journal of Thoracic Disease*.

[12] Bradley B., Branley H. M., Egan J. J. (2008). Interstitial lung disease guideline: the British Thoracic Society in collaboration with the Thoracic Society of Australia and New Zealand and the Irish Thoracic Society. *Thorax*.

[13] Ishioka S., Nakamura K., Maeda A. (2000). Clinical evaluation of idiopathic interstitial pneumonia and interstitial pneumonia associated with collagen vascular disease using logistic regression analysis. *Internal Medicine*.

[14] Uppal S., Diggle C. P., Carr I. M. (2008). Mutations in 15-hydroxyprostaglandin dehydrogenase cause primary hypertrophic osteoarthropathy. *Nature Genetics*.

[15] Kozak K. R., Milne G. L., Bentzen S. M., Yock T. I. (2012). Elevation of prostaglandin E_2_ in lung cancer patients with digital clubbing. *Journal of Thoracic Oncology*.

[16] Dackor R. T., Cheng J., Voltz J. W. (2011). Prostaglandin E_2_ protects murine lungs from bleomycin-induced pulmonary fibrosis and lung dysfunction. *American Journal of Physiology–Lung Cellular and Molecular Physiology*.

[17] Lappi-Blanco E., Kaarteenaho-Wiik R., Maasilta P. K., Anttila S., Pääkkö P., Wolff H. J. (2006). COX-2 is widely expressed in metaplastic epithelium in pulmonary fibrous disorders. *American Journal of Clinical Pathology*.

[18] Shiraishi K., Jinta T., Nishimura N. Digital clubbing is associated with higher serum KL-6 levels and lower pulmonary function in patients with interstitial lung disease. http://www.atsjournals.org/doi/pdf/10.1164/ajrccm-conference.2016.193.1_MeetingAbstracts.A5006.

